# Chinese students’ L2 classroom engagement in college: Perceived teacher support, value beliefs and self-monitoring

**DOI:** 10.1371/journal.pone.0356390

**Published:** 2026-08-17

**Authors:** Shuai Zheng, Yi Liu, Xi Peng, Dan Li

**Affiliations:** 1 School of Foreign Studies, University of Science and Technology Beijing, Beijing, People’s Republic of China; 2 College of Foreign Languages and Cultures, Chengdu University, Chengdu, People’s Republic of China; Guangdong University of Foreign Studies, CHINA

## Abstract

Based on an integrated framework of the Self-Determination Theory and the Expectancy-Value Theory, this study investigated the link between students’ perceived teacher support and their L2 classroom engagement via two mediators: students’ value beliefs of English learning and metacognitive self-monitoring with a sample of 377 college students from a Chinese university. With gender, age and onset age of English learning under control, path analyses showed that perceived teacher support directly predicted L2 classroom engagement, and the link between them was also positively mediated by either students’ value beliefs or self-monitoring. Moreover, this study also revealed that the indirect effect of perceived teacher support on L2 classroom engagement was significant through the serial mediation of students’ value beliefs and then self-monitoring. These findings suggest that in the context of L2 learning, higher level of perceived teacher support can promote college students’ L2 classroom engagement by stimulating students’ value beliefs of L2 learning and strengthening their metacognitive self-monitoring, which provides practical implications to language teaching activities in the classroom setting.

## Introduction

Thanks to the global status of the English language, many countries have integrated compulsory English courses across various stages of their educational systems. In China, English has been a compulsory subject for decades, normally spanning from early elementary school through the first two years of college. For many Chinese students, learning English as a second language (L2) represents a prolonged, effort-intensive journey heavily shaped by high-stakes tests during the primary and secondary phases, such as “*Gaokao*”, the National College Entrance Examination. Although high-stakes assessments persist at the tertiary level — most notably the College English Test Band 4 (CET-4) for non-English majors, a mandatory prerequisite for college graduation at many Chinese universities — the nature of college-level English learning shifts significantly away from the rigid, teacher-directed coaching of secondary school due to the nature of college education. With an increasing number of universities decoupling CET-4 from graduation requirements in recent years, college English learning has become more self-determined, interest-oriented, and motivation-driven. Consequently, understanding how to effectively foster and sustain student engagement in the college L2 classroom has attracted growing scholarly attention in recent years.

The Expectancy-Value Theory (EVT; [1,2]) is often applied to account for the motivational processes behind individual’s achievement-related outcomes [3,4], which suggests that achievement motivation stems from both people’s expectancy for future success on a task and their perceptions of task values [5], and these expectancy and perceptions are termed as value beliefs by the EVT [1]. In the context of L2 learning, college students’ learning motivation may be closely related to their evaluation and perceptions of the values of English learning and future learning outcomes, thus influencing their actions and behaviours in the L2 classroom, i.e., whether to actively engage in the L2 classroom, which will further have an impact on their L2 achievement, or final attainment.

However, college students’ value beliefs of English learning do not emerge in a vacuum, and it may be influenced or shaped by the beliefs and attitudes of other people around them. In college, students are closely connected with their teachers, and previous research indicates that a healthy academic environment where students are able to receive necessary help from teachers will promote their development (e.g., [6,7]. According to the Self Determination Theory (SDT), an individual’s motivation and self-initiated behaviours will be stimulated once their basic psychological needs for autonomy, competence and relatedness are satisfied [8–10]. In the case of L2 learning, teacher support may serve as the environmental nutrients that the students need, and the satisfaction of their psychological needs will further motivate them to get more engaged in the L2 classroom. Nevertheless, how college students’ perceived teacher support would specifically influence their value beliefs of English learning has not been fully investigated, and how and to what extent their L2 learning motivation driven by these value beliefs would in turn contribute to L2 classroom engagement also need further exploration.

In addition, previous literature presented positive evidence that metacognitive factors are actively involved in L2 learning, for example, self-efficacy [11], self-concept [12] and self-regulation [13]. As a highly conscious process, L2 learning is also a process of self-monitoring during which L2 learners constantly observe their real-time thinking and actions and regulate their behaviours. Though it seems to be a fact that metacognitive self-monitoring is linked to L2 learning, few empirical studies, to our knowledge, have provided solid evidence. And it is not yet clear as well whether self-monitoring is positively related to perceived teacher support and students’ value beliefs of L2 learning in the classroom.

Based on an integrated framework of the SDT and EVT, this study intends to investigate the relationships among students’ perceived teacher support, value beliefs of L2 learning, metacognitive self-monitoring and L2 classroom engagement in the Chinese context of non-English majors.

## Literature review

### Perceived teacher support and L2 classroom engagement

Kuh et al. [14] regard student engagement as the time and energy students invest into educational activities, whereas Wang, Henry and Degol [15] provide a more detailed definition, explaining it as students’ qualitative interactions or involvement with learning activities, processes and contexts. As for L2 engagement, it can be defined as the heightened involvement in language learning [16]. Different studies may adopt different definitions of L2 engagement with respect to varied research purposes and aims, and in the present study, L2 engagement is confined to students’ involvement and participation in L2 classroom activities and their interactions with the teacher and classmates (hereafter L2 classroom engagement), because language learning is a socializing process that involves dynamic interactions [17] and the classroom is the best place to observe such a process. Though varied in definition, it is commonly acknowledged that student engagement is multifaceted. Fredricks, Blumenfeld and Paris [18] proposed three key components of engagement: behavioural, cognitive and emotional engagement. And these three components characterize how students act, think, and feel in the context of L2 classroom instruction [19].

Similar to L2 engagement, teacher support is also a multidimensional construct that encompasses emotional support [20], academic instruction [21], constructive feedback [22], etc. Perceived teacher support refers to the extent to which students believe they can depend on the help or assistance offered by their teachers [23]. Many researchers and scholars have placed key emphasis on the role of perceived teacher support in school settings, suggesting that more support and guidance from the teacher enable students to be more interested or engaged in classroom activities [24,25].

Theoretically, the positive correlation between perceived teacher support and classroom engagement can be substantiated by the SDT. According to the SDT, individuals thrive when they perceive their social environments as actively supporting their basic psychological needs, during which process teacher support plays a crucial role. In the L2 learning literature, the relationship between perceived teacher support and L2 engagement has been studied widely. By using observational evaluation methods, Jang et al. [26] found that teacher-provided autonomy and structure positively predicted students’ behavioural engagement, and the study conducted by Zhou et al. [27] further confirmed that an autonomy supportive environment and a controlling one will lead to qualitative differences in students’ L2 classroom engagement. In a more recent questionnaire-based study, Vo et al. [28] also revealed that how students perceive their language teachers to be responsive to their emotions is closely related to their L2 classroom engagement. Therefore, based on the existing literature, the following baseline hypothesis is proposed:

**H1**: College students’ perceived teacher support positively predicts their L2 classroom engagement.

### Relationship between value beliefs and L2 classroom engagement

Although positive teacher support helps to establish the prerequisite for an effective L2 learning environment, it is the students themselves that is the decisive factor in successful L2 learning, and recent L2 learning research has witnessed the increased attention on the internal psychological factors within the L2 learners. Task value belief is a key concept in the EVT [29,30], an influential framework that guides much research on students’ motivation that influences their performance and achievement-related choices [31]. According to the EVT, task value contains 4 major components, namely, attainment value, intrinsic value, utility value, and cost [29,32]. The former three as a whole are termed as value beliefs, each of which may bring an effect to an individual’s motivation behind a task.

Recent years have witnessed a growing body of research that reported the positive effects of students’ individual or collective task values on L2 engagement. For instance, Vo [19] found that Vietnamese students’ perceived utility value of L2 learning plays an essential role in active L2 classroom engagement, and Man and Zhang [33] revealed that Chinese high schoolers’ extrinsic value composed of utility and attainment value and their intrinsic value both significantly predicted students’ L2 engagement. Similar findings on the association between task value and L2 engagement were also reported in Hoi [34], Vo et al. [28], Vo et al. [31]. However, in a study targeting college students’ attainment value and interest value, Man [35] only found attainment value a positive predictor of L2 engagement, suggesting that the effects of the three task values are not equal and may vary across different L2 age groups.

As L2 learners’ motivation behind L2 learning is a comprehensive, complex and dynamic continuum, we followed Vo et al. [36] by combing the three task values as a single construct — subjective task value beliefs — to represent the collective psychological force of students’ internalised, autonomous valuation of the L2 learning task, instead of treating them as independent variables in the present study. Based on the findings of majority research, we propose the following hypothesis:

**H2**: College students’ value beliefs of L2 learning positively predicts their L2 classroom engagement.

### Relationship between metacognitive self-monitoring and L2 classroom engagement

Beyond external environment and internal motivation, students’ cognitive and behavioural manifestations in the classroom are inherently governed by their execution of self-regulatory strategies, and a number of studies have probed the relationship between self-regulation and L2 engagement (see [37] for an overview), for example, Zhou and Hiver [38] suggest that self-regulated learning strategies function as an important antecedent to L2 learners’ engagement in the writing class. Within the broad framework of self-regulated learning, metacognition is a critical component. It is defined by Flavell [39] as “thinking about thinking”, and is regarded as a predictor of adaptive thought and action [40]. Successful individuals are capable of taking advantage of their own thought and actions, through which self-awareness, self-determination and self-direction can be developed [41]. In the literature of L2 learning, a number of factors related to metacognition have been examined, such as anticipatory planning and metacognitive task-monitoring [41].

As an essential component of metacognition, metacognitive monitoring involves the perception and assessment of one’s own cognitive activity [42], yet research on the effect of metacognitive monitoring on L2 classroom engagement is relatively limited. In order to distinguish interpersonal monitoring [43] and intrapersonal monitoring [44], we adopt the term “metacognitive self-monitoring (‘self-monitoring’ for short)” in this study and define it as the cognitively self-initiated activities to monitor one’s cognitive engagement in the L2 classroom. Metacognitive monitoring is the deliberate, ongoing process wherein learners track, evaluate, and regulate their own cognitive processing and attention during learning tasks [39], and hypothetically, students of higher self-monitoring ability will be more engaged in classroom activities and tasks, while students who are not good at monitoring themselves may be more easily distracted or disengaged in the L2 classroom. Based on this, we propose the following hypothesis:

**H3**: College students’ metacognitive self-monitoring positively predicts their L2 classroom engagement.

### Mediating role of value beliefs and self-monitoring

Beyond the direct pathways, L2 classroom engagement is believed to be governed by a sequential, multi-stage causal chain where distal environmental factors are internalised into proximal cognitive and behavioural regulations. Previous research has validated that teacher support significantly influences students’ L2 learning motivation, for example, by targeting undergraduate English L2 learners in China, Zhang and Hu [45] found that L2 motivation positively mediates the link between perceived teacher support and English classroom engagement. As students’ value beliefs are directly linked with their motivation and achievement choices in the EVT framework [46], the following hypothesis is proposed:

**H4a**: Value beliefs of L2 learning mediates the relationship between perceived teacher support and L2 classroom engagement.

In addition, it is revealed that teacher support can equip students with self-regulatory skills that enhance their ability to manage emotions, set goals, and navigate challenges (e.g., [47]), which are closely related to self-observation, self-control and self-instruction. In the L2 classroom, it is expected that students may be more conscious of their actions and monitor themselves more frequently when they find the teachers academically and emotionally supportive, and in turn be more engaged in classroom activities. Hence, the following hypothesis is proposed:

**H4b**: Self-monitoring mediates the relationship between perceived teacher support and L2 classroom engagement.

Besides the potential mediating role of value beliefs and self-monitoring between perceived teacher support and L2 classroom engagement, there might be a serial mediation chain via value beliefs and then self-monitoring. In a study on massive open online course (MOOC) learners, Zhu and Doo [48] paid special attention to students’ self-directed learning abilities and found that students’ motivation positively predicted self-monitoring. Considering that value beliefs of L2 learning fundamentally serve as the core of L2 learning motivation, it can be predicted that students’ value beliefs may be positively associated with self-monitoring in the L2 classroom, specifically, higher value beliefs may stimulate more self-monitoring, which will lead to more active and conscious engagement in class, hence the following serial mediation hypothesis:

**H4c**: The link between perceived teacher support and L2 classroom engagement is serially mediated by value beliefs and then self-monitoring.

**Fig 1:**
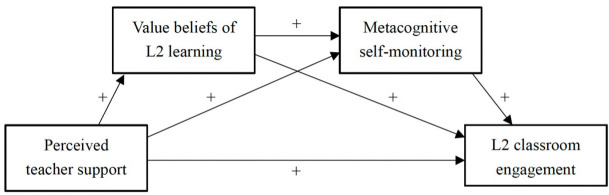
The hypothesised mediation model.

In the present study, we believe that the integration of the SDT and the EVT can precisely capture the relationships between the four variables, forming a pathway as “Context→Valuation→Regulation→Action”. As Fig 1 shows, within this theoretical continuum, perceived teacher support serves as the initial environmental factor that fulfills students’ basic psychological needs, based on the SDT. Then the psychological and academic support students receive from the teacher and their interaction with the teacher can lead to the internalization and formulation of positive value beliefs of L2 learning, i.e., utility value, attainment value and interest value, as proposed by the EVT. Crucially, these value beliefs do not act as static psychological outcomes; rather, according to the SDT, they serve as the dynamic motivation that mobilises individuals’ tactical self-regulatory and self-monitoring ability. Since self-monitoring (e.g., tracking attention, evaluating comprehension) is an intensive process that demands significant cognitive resources [49], the more the students perceive L2 learning valuable, the more they will actively monitor themselves in the L2 classroom, so as to remain attentive and be fully engaged in learning activities.

## Methods

### Participants

Data were collected using convenience sampling from first-year and second-year non-English majors at a university in China. The period of participant recruitment and data collection lasted a month, from June 1^st^ 2025 to June 30^th^ 2025. All of the participants had the English subject back to their primary and secondary education, and during the time of data collection, they were attending weekly compulsory English class at university entitled *College English*, which is an integrated course by nature and lasts for 2 years, covering essential components in English like listening, reading, writing, speaking and translating skills. Students were explained the purpose and procedure of this study before data collection, and were then invited to participate anonymously on a voluntary basis. All the participants gave their written consent via a separate online questionnaire containing the consent form before filling out the formal questionnaire.

A total of 428 students completed and submitted the questionnaire, of which 51 (11.92%) were considered invalid and excluded from further analysis. In the remainder of the questionnaires (N = 377, 88.08%), the number of freshman students was 97, and the other 280 were sophomores. Male participants accounted for 48.28% (N = 182) of the total, with an average age of 19.80 (SD = 1.043), and their female counterparts took up 51.72% (N = 195), with an average age of 19.69 (SD = 0.902).

### Instruments

This study adopted an online questionnaire via *Wenjuanxing* (a platform specially designed for online surveys in Chinese mainland) to collect data. The questionnaire comprised two parts: the first part was about students’ demographic information, such as gender, age, grade, etc., and the second part consisted of four 5-point Likert scales measuring each of the 4 variables in this research, i.e., students’ perceived teacher support, value beliefs, metacognitive self-monitoring, and L2 classroom engagement (provided in S1 Appendix). Students could use the link provided to access the questionnaire which was written in Chinese.

#### Perceived teacher support.

Previous literature regards teacher support as a multifaceted factor in students’ academic activities whose major components mainly include emotional support, agentive support and cognitive support (e.g., [50]) in questionnaire scales. Emotional support refers to the positive attention, care, and emotional connection that teachers offer to students, as well as the support to deal with pressure, challenges and negative emotions [51]. Agentive support is the sufficient freedom and support usually showcased in the assignment of tasks, the selection of teaching/learning materials and the training on problem-solving skills [52]. A number of studies did not make a clear distinction between agentive support and cognitive support, and combined them as one component termed as cognitive agentive support to measure (e.g., [53]). But there are also scholars suggesting that cognitive support is the offering of academic challenges and the stimulation of students’ curiosity and motivation for learning [54]. Taking into consideration of the educational system in China and the characteristics of Chinese students, Chai and Gong [55] made the 17-item scale (Cronbach’s α = 0.92) for students’ perceived teacher support in math class, of which three constructs are measured, i.e., emotional support, agentive support and cognitive support. Guided by our research aims, we adopted this scale and made corresponding adaptations by changing the subject name from “math” into “English”. In this scale, seven items were used to measure emotional support, for example, “the English teacher likes me”. Five items were for agentive support, and a sample item was “the English teacher tries to make us understand the importance of learning English”. Another five items were for cognitive support, for instance, “the English teacher provides us with useful English learning methods and skills”. Reliability analysis showed that the scale had a high internal consistency (Cronbach’s α = 0.968). The results of the confirmatory factor analysis (CFA) demonstrated that the scale exhibited a highly satisfactory and robust model fit to the current data: *χ*^*2*^ (116) = 161.235, CFI = 0.986 > 0.9, TLI = 0.984 > 0.9, RMSEA = 0.032 < 0.08 (90% CI [0.019, 0.043]), SRMR = 0.029 < 0.08 [56].

#### Value beliefs of English learning.

Students’ value beliefs of English learning were measured by a 12-item scale adapted from Part et al. [5] from three aspects. Four items assessed students’ attainment value, i.e., the extent of the importance of successful completion of a task (e.g., Is the amount of effort it will take to do well in your English course worthwhile to you?). Intrinsic value, the interest in and joy from a task, were gauged by 4 items, for instance, “Learning the material covered in my English course is enjoyable”. Lastly, the utility value, the perceived usefulness of a task for future development, was estimated by another 4 items, and a sample item was “What I learn in my English course will be useful for me later in life”. The internal consistency (Cronbach’s α) for the value beliefs scale in this study was 0.94. The results of the CFA demonstrated that the scale exhibited a highly satisfactory and robust model fit to the current data: *χ*^*2*^ (51) = 126.262, CFI = 0.964 > 0.9, TLI = 0.954 > 0.9, RMSEA = 0.063 < 0.08 (90% CI [0.049, 0.076]), SRMR = 0.048 < 0.08 [56].

#### Metacognitive self-monitoring.

Students’ metacognitive self-monitoring was measured by a 6-item scale developed by Hiver et al. [41], which targets the specific metacognitive awareness and self-monitoring process of language learners in the classroom setting. A sample item is “In the language classroom, I am aware of what I am doing as I learn”. Internal reliability analysis showed that one item (In the language classroom, I care very little about how others react to me) should be removed from the scale because of a low corrected item-total correlation for this item (−.563) and a Cronbach’s α at 0.66 < 0.7. In addition, this item was weakly correlated with the other five items respectively (0.3 < |rs| < 0.5). The deletion of this item was supported by the data for having an increase in internal consistency (Cronbach’s α = 0.893). Hence, there were 5 items left for this self-monitoring scale. The results of the CFA demonstrated that the scale exhibited a highly satisfactory and robust model fit to the current data: *χ*^*2*^ (5) = 10.768, CFI = 0.986 > 0.9, TLI = 0.971 > 0.9, RMSEA = 0.055 < 0.08 (90% CI [0.019, 0.043]), SRMR = 0.022 < 0.08 [56].

#### L2 classroom engagement.

Students’ L2 classroom engagement was measured by a 12-item scale borrowed from Vo [19]. The original scale by Hiver et al. [41] consists of 24 items, measuring students’ behavioural, cognitive and affective engagement in L2 learning activities. Vo [19] selected 12 items from the original scale and made slight adaptations in terms of wording, by adding phrases like “in my English class” to each item, and used the simplified version to test Vietnamese students’ L2 English engagement in the classroom. In consideration of time, efficiency and accuracy, we adopted the simplified version of the scale Vo [19] adapted, and believe that it specifically targets L2 English engagement in the classroom setting. In the scale, four items were for the measurement of students’ behavioural engagement, such as “When I am in my English class, I pay attention and listen carefully”. Another four items focused on the aspect of cognitive engagement, for example, “In my English class, I think about different ways to solve a problem”. Finally, affective engagement was evaluated by the last four items, and a sample item was “I feel good when I am in my English class”. The internal consistency (Cronbach’s α) for the L2 classroom engagement scale in this study was 0.969. The results of the CFA demonstrated that the scale exhibited a highly satisfactory and robust model fit to the current data: *χ*^*2*^ (49) = 132.865, CFI = 0.960 > 0.9, TLI = 0.946 > 0.9, RMSEA = 0.067 < 0.08 (90% CI [0.054, 0.081]), SRMR = 0.034 < 0.08 [56].

### Data analysis

#### Covariates.

Previous research has suggested the gender effect on L2 learning and achievement in various ways, for example, the use of metacognitive strategies and English language proficiency (e.g., [57]) and L2 learning motivation (e.g., [58]). Besides, the age effect has long been the focus of language acquisition and L2 learning, and a recent study indicated that age was closely linked with environmental supports, cognitive abilities, and motivation for language learning [59]. In order to minimize the effect of gender and age-related effects, we controlled gender, age (range: 17–24; Mean = 19.74; SD = .97) and onset age of formal English learning (hereafter OAE; range: 5–15; Mean = 9.95; SD = 2.35) as covariates in data analysis. As a categorical variable, gender was turned into a dummy variable, with “male” coded as 1 and “female” 0. While age and OAE are continuous variables, the values of each were converted into standard scores (z-scores) with a mean average of 0 and an SD of 1.

#### Data analysis procedure.

The raw data were exported into an Excel spreadsheet from the online survey platform, and invalid samples were excluded before formal statistical analysis which were carried out in five steps. First, standardised factor loadings, composite reliability, average variance extracted (AVE) values and the square roots of AVE values of the latent constructs were calculated to evaluate the reliability and validity of the instruments, along with a correlation analysis between the constructs. Then, Harman’s single-factor test and the unmeasured latent method construct (ULMC) approach were conducted respectively to evaluate the potential threat of common method bias. Next, descriptive analysis and Pearson partial correlation analysis (with gender, age and OAE controlled) among the four manifest variables were performed via SPSS 29.0.1, so as to gain a preliminary insight into the relationships between the constructs. Following that, a multicollinearity assessment was executed to evaluate the stability of the four manifest composites. Finally, based on our hypothesised mediation model, model fit was tested using maximum likelihood estimation in Mplus 8.1.6, and corresponding path analyses were conducted to examine the mediation effects of value beliefs and self-monitoring between perceived teacher support and L2 classroom engagement. To establish robust standard errors and evaluate the operational efficacy of the alternative indirect strings, a standard non-parametric bootstrapping procedure based on 5000 random resamples was strictly implemented. If the bias-corrected 95% confidential interval (CI) does not contain 0, the respective indirect effect is validated as statistically significant [60].

## Results

### Construct reliability and validity

To evaluate the reliability of the instruments, we examined the standardised factor loadings and composite reliability (CR) of each scale, alongside the CFA results of the scales reported in the Methods section. As Table 1 shows, the standardised factor loadings were all above the threshold of 0.5, with the majority above the conservative threshold of 0.7; the CR values were all above the cutoff benchmark of 0.70, indicating sufficient measurement reliability [61].

**Table 1:** Standardised factor loadings, CR and AVE values of the instruments.

Construct	Item	Standardised Factor Loadings (λ)	Composite Reliability (CR)	Average Variance Extracted (AVE)
PTS-Emotional	7	0.854–0.934	0.963	0.811
PTS-Agentive	5	0.879–0.946	0.960	0.828
PTS-Cognitive	5	0.892–0.953	0.965	0.865
VB-Attainment	4	0.589–0.892	0.863	0.617
VB-Intrinsic	4	0.852–0.927	0.935	0.783
VB-Utility	4	0.711–0.896	0.913	0.720
SM	5	0.735–0.848	0.895	0.630
CE-Behavioural	4	0.684–0.925	0.919	0.742
CE-Affective	4	0.860–0.926	0.947	0.816
CE-Cognitive	4	0.716–0.911	0.915	0.731

Note: PTS-perceived teacher support, VB-value beliefs, SM-self-monitoring, CE-classroom engagement.

For the validity of the instruments, we firstly calculated the average variance extracted (AVE) values to evaluate the convergent validity of the latent constructs. Following Fornell and Larcker [62], an AVE value that is above 0.50 is typically interpreted as evidence that the items capture a meaningful portion of shared variance, and the AVE values for all latent constructs exceeded this benchmark (refer to Table 1).

Following that, discriminant validity was evaluated to guarantee that all the latent constructs are psychometrically independent. According to Fornell and Larcker [62], discriminant validity is established when the square root of the AVE value for each construct is greater than its correlation coefficients with any other construct. As presented in Table 2, all the square roots were higher than the corresponding correlation coefficients.

**Table 2:** Results of discriminant validity and correlation analysis.

Construct	1	2	3	4	5	6	7	8	9	10
1. PTS-Emotional	**0.901**	—	—	—	—	—	—	—	—	—
2. PTS-Agentive	0.742	**0.910**	—	—	—	—	—	—	—	—
3. PTS-Cognitive	0.676	0.831	**0.930**	—	—	—	—	—	—	—
4. VB-Attainment	0.400	0.347	0.313	**0.785**	—	—	—	—	—	—
5. VB-Intrinsic	0.608	0.479	0.433	0.605	**0.885**	—	—	—	—	—
6. VB-Utility	0.514	0.419	0.369	0.731	0.702	**0.849**	—	—	—	—
7. SM	0.449	0.347	0.321	0.518	0.545	0.517	**0.794**	—	—	—
8. CE-Behavioural	0.557	0.436	0.400	0.557	0.620	0.584	0.750	**0.861**	—	—
9. CE-Affective	0.579	0.443	0.409	0.545	0.636	0.580	0.745	0.850	**0.903**	—
10. CE-Cognitive	0.553	0.424	0.375	0.550	0.591	0.579	0.774	0.854	0.871	**0.882**

Note: PTS-perceived teacher support, VB-value beliefs, SM-self-monitoring, CE-classroom engagement. Diagonal values (in bold) represent the square roots of the AVE values.

Taken together, the results showed sufficient reliability and robust validity of the instruments.

### Common method bias

To assess the potential threat of common method bias (CMB), Harman’s single-factor test was conducted using an unrotated exploratory factor analysis (EFA). The results revealed that the first factor accounted for 49.093% of the total variance, which was below the critical threshold of 50% [63], indicating that a single dominant factor did not emerge in the present study.

Considering that the value derived from Harman’s single-factor test was relatively close to the cutoff threshold, we implemented the more rigorous unmeasured latent method construct (ULMC) approach to further scrutinize the potential threat of CMB [63]. First, a baseline measurement model (Model 1) was estimated by incorporating all underlying item-level indicators across the four constructs: perceived teacher support, value beliefs, self-monitoring, and L2 classroom engagement. Then, an alternative nested model (Model 2) was constructed by introducing an unmeasured common latent factor which allowed all observed indicators to load simultaneously onto it while its variance was constrained to be strictly orthogonal to all substantive factors. The model fit indices for the two models were compared against each other as follows: ΔCFI = CFI_Model 2_ (0.954) – CFI_Model 1_ (0.949) = 0.005 < 0.01, ΔRMSEA = RMSEA_Model 1_ (0.043) – RMSEA_Model 2_ (0.041) = 0.002 < 0.015 [64], and the comparison indicated that the addition of the common latent factor did not significantly improve the model fit, confirming that CMB was not a major concern to the validity of the findings in this study.

### Descriptive statistics and correlation analysis

To examine the central tendency and distributional properties of each composite construct, we calculated the values for the mean, standard deviation (SD), skewness (SK) and Kurtosis (Kur) of the four constructs. The absolute values of skewness and kurtosis all fell into recommended ranges (|SK| < 2; |Kur| < 7), suggesting that the data were approximately normally distributed. Table 3 presents the descriptive statistics and Pearson partial correlations among the four variables.

**Table 3:** Descriptive statistics and correlation analysis.

	Mean	SD	SK	Kur	1	2	3	4
1. PTS	4.244	0.589	−0.165	−0.858	1			
2. VB	3.789	0.660	−0.089	0.247	0.541^***^	1		
3. SM	3.609	0.687	0.164	0.145	0.411^***^	0.595^***^	1	
4. CE	3.701	0.712	0.198	−0.158	0.535^***^	0.687^***^	0.789^***^	1

Note: PTS-perceived teacher support, VB-value beliefs, SM-self-monitoring, CE-classroom engagement.

***P < 0.001.

As Table 3 shows, the correlation analysis revealed that perceived teacher support, value beliefs of English learning, metacognitive self-monitoring and L2 engagement were significantly positively linked with each other.

### Multicollinearity assessment

To verify the interpretability and stability of the model, we conducted a multicollinearity assessment for the independent variables by evaluating Tolerance and Variance Inflation Factor (VIF). As shown in Table 4, the tolerance value for each of the constructs exceeded 0.1, and the VIF values were all below the recommended cutoff of 5 [61], based on which it can be confirmed that multicollinearity did not pose a threat to the stability or estimation of our structural paths.

**Table 4:** Results of multilinearity assessment.

Construct	Tolerance	VIF
PTS	0.695	1.438
VB	0.541	1.850
SM	0.635	1.576

Note: PTS-perceived teacher support, VB-value beliefs, SM-self-monitoring.

### Path analysis

To evaluate the hypothesised mediation model, composite scores of the four variables were used to estimate the interaction effects in a path analysis model, with participants’ gender, age, and OAE controlled as covariates. The path analysis yielded a fully saturated model: *χ*^*2*^ (0) = 0, CFI = 1.00 > 0.9, TLI = 1.00 > 0.9, RMSEA = 0.00 < 0.08 (90% CI [0.00, 0.00]), SRMR = 0.00 < 0.08 [65]. As the statistical model is fully saturated, evaluating global model fit would be uninformative. Instead, we evaluated the model based on the estimated path coefficients and bootstrap confidence intervals for the mediation effects.

**Fig 2:**
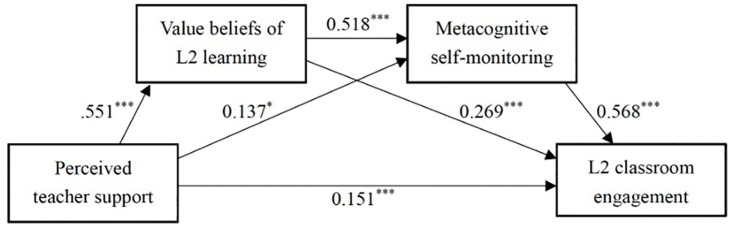
Diagram of mediation paths.

The path analysis revealed positive correlations between the four variables, as summarised in Fig 2 and Table 5. For the direct paths, the results showed that perceived teacher support, value beliefs and self-monitoring all significantly and positively predicated L2 classroom engagement (*β* = 0.151, 95% CI [0.084, 0.217]; *β* = 0.269, 95% CI [0.170, 0.368]; *β* = 0.568, 95% CI [0.469, 0.659]), proving the validity of H1, H2 and H3 respectively. For the mediated paths, the analysis suggested that the link between perceived teacher support and L2 classroom engagement was positively mediated by either value beliefs (*β* = 0.148, 95% CI [0.091, 0.210]) or self-monitoring (*β* = 0.078, 95% CI [0.017, 0.143]) independently, so both H4a and H4b were confirmed. Lastly, when both value beliefs and self-monitoring were added into the path sequentially, the result revealed that the link between perceived teacher support and L2 classroom engagement was serially mediated by the two variables (*β* = 0.162, 95% CI [0.116, 0.210]), hence H4c was also confirmed.

**Table 5:** Mediation effects of VB and SM.

Effect	Path	*β*	Bootstrap 95% CI		Percentage (%)
			LLCI	ULCI	
Total effect	PTS → CE	0.539	0.459	0.613	100%
Direct effect	PTS → CE	0.151	0.084	0.217	28.0%
Indirect effect	PTS → VB → CE	0.148	0.091	0.210	27.5%
	PTS → SM → CE	0.078	0.017	0.143	14.5%
	PTS → VB → SM → CE	0.162	0.116	0.210	30.1%

Note: PTS-perceived teacher support, CE-classroom engagement, VB-value beliefs, SM-self-monitoring.

## Discussion

Drawing upon an integrated framework of the SDT and EVT, this study constructed and tested a serial mediation model specifying the pathway from teacher support to L2 classroom engagement via L2 value beliefs and self-monitoring. The total effect of teacher support on L2 classroom engagement was substantial (*β* = 0.539, 95% CI [0.459, 0.613]), indicating that teacher support is a powerful predictor of students’ classroom participation, which is consistent with previous studies (e.g., [27,28]). Critically, the decomposition of the total effect into direct and indirect components reveals a nuanced picture of the underlying mechanisms, showcased by three mediation paths.

### Mediation of value beliefs

Among the indirect pathways, the serial mediation through value beliefs served as a strong contributor, accounting for 27.5% of the total effect, revealing the crucial role of learning motivation through which external teacher support influences students’ L2 engagement by shaping their value beliefs of L2 learning.

Based on the SDT, the satisfaction of individuals’ basic psychological needs for autonomy, competence, and relatedness increases their motivation and participation [8–10]. In the L2 classroom context, teacher support serves as the primary environmental resource that fulfil students’ psychological needs and stimulate their motivation. For relatedness, L2 learners have constant contact in the classroom with their English teacher who may be seen as an important attachment figure with whom they feel closely connected [66]. The concept of “competence” refers to the capacity that students themselves can successfully meet challenges and master new skills, which can be satisfied from teacher’s agentive and cognitive support. And as college learning is highly self-dependent in nature, students can also enjoy a high degree of autonomy regarding their own learning. Therefore, once students’ basic psychological needs are guaranteed, they may regard the teacher as role model and adopt the concepts and values they hold.

As L2 learners themselves, language teachers clearly know the crucial role of English learning in a globalised world and the practical value of good English skills for personal and career development, and know the necessity and importance to pass the same value beliefs onto students via carefully designed classroom activities and discussions, through which students’ value beliefs will be shaped and strengthened explicitly or implicitly, which further contributes to their L2 classroom engagement. In China, where the culture has been deeply influenced by Confucianism, teachers are usually regarded as know-all experts [67] and authority, and students may be more easily influenced by the teacher’s behaviours and value beliefs.

Therefore, a healthy teacher-student relationship not only enables students to feel connected, gain confidence and develop competence, but also helps them establish healthy and positive value beliefs that motivate L2 engagement.

### Mediation of self-monitoring

This study also confirmed the mediating role of metacognitive self-monitoring between perceived teacher support and L2 classroom engagement, underscoring the role teachers play in promoting students’ self-monitoring ability.

In psychology, self-monitoring is a part of learners’ metacognition which refers to their self-awareness and regulation of their cognitive processes [39,68]. As L2 class is usually instructed in the target language, students may find it a highly-anxious and cognitive demanding task and are more easily to be distracted by other irrelevant stuff, so patient, encouraging teacher support may prompt them to deploy active cognitive energy to monitor their attention in real time and regulate their behaviours in the L2 classroom. Besides, the positive link between self-monitoring and L2 classroom engagement suggests that students of higher self-monitoring capacity engage more in L2 learning activities. Those who are good at monitoring their cognitive processes are apt to notice whether they are focused or distracted in class earlier and more easily, thus helping them maintain their attention or take corresponding strategies to drag them back to the right track when distracted.

Although self-monitoring is an essential part of self-regulation and a few studies have proved the positive association between self-regulation and learning achievements (e.g., [13]), we believe that self-monitoring and self-regulation are two distinct construct, as self-regulation is much broader in scope and self-monitoring is situated at the front end of the self-regulation spectrum. In this regard, this study provides empirical evidence of the relationship between self-monitoring and L2 classroom engagement, which is less researched in L2 motivation literature.

### Serial mediation of value beliefs and self-monitoring

The path analysis results showed that the serial mediation of value beliefs and metacognitive self-monitoring contributed the most to the indirect effects (30.1%), surpassing both the value belief-mediated (27.5%) and the self-monitoring-mediated pathways (14.5%). This finding underscores the critical theoretical insight that teacher support exerts its most powerful influence on classroom engagement not through a single psychological mechanism, but through a motivational-cognitive cascade: teacher support first fosters L2 value beliefs, which in turn promote self-monitoring, ultimately leading to enhanced classroom participation. The whole chain of the four variables in this study can be interpreted from the perspective of the integrated framework of SDT and EVT.

As previously discussed, the link between perceived teacher support and value beliefs finds its theoretical foundation in the SDT, as teacher support can drive self-initiated behaviour and motivation as long as students’ basic psychological needs are satisfied [8–10]. In terms of the link between value beliefs and self-monitoring, it is best explained by the EVT, which posits that individual’s task value beliefs comprising attainment value, utility value and intrinsic value are key determinants of achievement-related choices, persistence and effort expenditure [29]. Self-monitoring, as a metacognitive strategy, demands sustained attentional and executive resources. According to EVT’s cost-benefit logic, learners will invest these resources only when the perceived benefits of engagement (captured by value beliefs) outweigh the perceived costs. Hence, strong value-belief holders will invest more time and energy in self-monitoring, whereas those who possess less sense of value beliefs will naturally put less effort into monitoring themselves.

Lastly, the link between self-monitoring and L2 classroom engagement represents the convergence of the theoretical narratives established in the first two stages. Once value beliefs have been shaped and internalised, self-monitoring serves as the final mechanism that turns motivational energy into observable behavioral engagement. Proactive self-monitoring enables students to stay focused and track their comprehension constantly, yielding higher-quality engagement than passive attendance [69], and it can also enhance students’ sense of autonomy and control, as they are managing their own learning process, and this may in turn further enhance their motivation and promote self-monitoring and engagement.

Generally, the finding of the serial mediation effect in the present study not only provides empirical validation for an integrated SDT-EVT framework, but also sheds light on the relationship between L2 learning motivation and metacognitive self-monitoring which may provide new insight to L2 teaching and learning.

## Implications and limitations

The findings of this research can provide certain pedagogical implications for L2 learning and teaching. First, teachers should create a comfortable and reliable learning environment for students by providing them with enough trust, freedom and practical skills and knowledge so as to satisfy their psychological needs for relatedness, autonomy and competence, which are the prerequisite of motivation stimulation and subsequent investment in L2 learning.

Second, as value beliefs function as the critical cognitive engine in the mediation chain, language teachers should lay emphasis on the cultivation and construction of students’ value beliefs underpinning L2 learning motivation. Teachers may try to cultivate students’ interest in the L2 via diversified classroom activities and games, and guide students to form healthy and positive utility and attainment values about L2 learning via carefully chosen texts/contents and well-designed tasks and discussions.

Besides, given that teacher support can positively predict students’ self-monitoring in the classroom, teachers may pay special attention to the language they use to make sure that it is easy to understand for the majority, and that the instructions they give for tasks and activities are clear and easy to be detected by students.

Nonetheless, there are some limitations in the present study. First, the data came from students studying at the same university in China, so the universality of the results shall be further tested in future research by comparing data from universities of different levels, different places and even different countries. Second, convenience sampling was used in questionnaire data collection, which can be improved by using more sophisticated sampling methods so that the results can be more robust and representative. Lastly, this research relied on a cross-sectional design to test the mediation effects, and the cross-sectional nature of the data may bias the mediation estimates. Therefore, longitudinal designs and qualitative methods can be adopted in future research to provide deeper, descriptive insights into the underlying mechanisms.

## Conclusion

By analysing self-reported questionnaire data from a student sample of a Chinese university, the present study revealed the mediating roles of students’ L2 value beliefs and self-monitoring respectively between perceived teacher support and L2 classroom engagement, and crucially, it also validated the serial mediation effect of the two mediators. The findings not only add empirical evidence to the SDT and the EVT, but also shed light on the indispensable role of perceived teacher support in the L2 learning process and the significant effect of students’ value beliefs, a crucial manifestation of their L2 learning motivation.

## Supporting information

S1 Appendix Questionnaire items used in this study. (DOCX)
