# Supplementary material for: Chinese students’ L2 classroom engagement in college: Perceived teacher support, value beliefs and self-monitoring
Source: PLoS One. 2026 Aug 17;21(8):e0356390. doi: 10.1371/journal.pone.0356390 (PMC13480610; doi:10.1371/journal.pone.0356390)
Supplement: Supplementary file 1 [file pone.0356390.s001.docx]

**Appendix**

**Questionnaire items:**

**Perceived Teacher Support**

*Emotional support*

1. My English teacher likes me.
2. My English teacher knows me well and cares about me.
3. I find it easy to communicate with my English teacher.
4. My English teacher believes that I can do well in the College English course.
5. My English teacher often encourages me.
6. My English teacher considers me to be a hard-working student.
7. My English teacher is willing to spend time helping me.

*Agentive support*

8. Our English teacher allows us to give answers that differ from the standard when solving problems.
9. Our English teacher tries their best to help us understand the importance of learning English.
10. Our English teacher takes our opinions into consideration when preparing lessons.
11. Our English teacher provides us with sufficient time for self-study or independent thinking.
12. Our English teacher encourages us to raise questions about what we have learnt.

*Cognitive support*

13. Our English teacher frequently draws inferences from one example to another and extends the learning content.
14. Our English teacher provides us with useful English learning methods and skills.
15. Our English teacher poses a series of questions about the teaching content to guide us toward the answers step by step.
16. Our English teacher encourages and guides us to actively explore solutions to problems rather than telling us the answers too early.
17. Our English teacher assigns tasks of moderate difficulty rather than exercises that are too easy or too difficult.

**Value Beliefs of English Learning**

*Attainment value*

1. Is the amount of effort it will take to do well in your College English course worthwhile to you? (Not at all worthwhile – Not worthwhile – Somewhat not worthwhile – Somewhat worthwhile – Worthwhile – Very worthwhile)
2. I feel that, to me, being good at solving problems that involve English reasoning is... (Not at all important – Not important – Somewhat not important – Somewhat important – Important – Very important)
3. How important is it to you to get a good grade in your College English course? (Not at all important – Not important – Somewhat not important – Somewhat important – Important – Very important)
4. It is important for me to be someone who is good at solving problems that involve the use of English. (Not at all important – Not important – Somewhat not important – Somewhat important – Important – Very important)

*Intrinsic value*

5. In general, I find working on assignments/studying for College English course to be… (Very boring – boring – Somewhat boring – Somewhat interesting – Interesting – Strongly interesting)
6. How much do you like your College English course? (I dislike it very much – I dislike it – I somewhat dislike it – I somewhat like it – I like it – I like it very much)
7. Learning the material covered in my College English course is enjoyable. (Strongly disagree – Disagree – Somewhat disagree – Somewhat agree – Agree – Strongly agree)
8. The contents and knowledge taught in my College English course is interesting. (Strongly disagree – Disagree – Somewhat disagree – Somewhat agree – Agree – Strongly agree)

*Utility value*

9. How useful is your College English course for what you want to do after you graduate and go to work? (Not at all useful – Not useful – Somewhat not useful – Somewhat useful – Useful – Very useful)
10. How useful is your College English course for your daily life outside school? (Not at all useful – Not useful – Somewhat not useful – Somewhat useful – Useful – Very useful)
11. What you learn in your College English course will be useful for you later in life. (Strongly disagree – Disagree – Somewhat disagree – Somewhat agree – Agree – Strongly agree)
12. Being good at English will be important when you get a job. (Strongly disagree – Disagree – Somewhat disagree – Somewhat agree – Agree – Strongly agree)

**Metacognitive Self-monitoring**

1. In the language classroom, I am aware of what I am doing as I learn.
2. I am in tune with others in the language classroom.
3. In the language classroom, I care very little about how others react to me. (R)
4. I pay attention to myself in relation to unfolding classroom events.
5. I know when my classroom language learning is on-target and when it is not.
6. I am aware of it when the language learner I am in the classroom does not match up with who I want to be as a language user.

**L2 engagement**

*Behavioral Engagement*

1. When I cannot understand in my English class, I stay focused until I do.
2. I keep trying in my English class even if something is hard.
3. I complete my English learning homework on time.
4. When I am in my English class, I pay attention and listen carefully.

*Emotional Engagement*

5. I look forward to my English class.
6. I enjoy learning new things about English in my English class.
7. I want to understand what I am learning in my English class.
8. I feel good when I am in my English class.

*Cognitive Engagement*

9. I go through the work for my English class carefully and make sure that it’s done right.
10. In my English class, I think about different ways to solve a problem.
11. I try to connect what I am learning in the English classroom to things I have learnt before.
12. I try to understand my mistakes in the English classroom when I get something wrong.

Note: items with (R) are negatively keyed and reverse coded.
